# Synthetic Protocells Interact with Viral Nanomachinery and Inactivate Pathogenic Human Virus

**DOI:** 10.1371/journal.pone.0016874

**Published:** 2011-03-01

**Authors:** Matteo Porotto, Feng Yi, Anne Moscona, David A. LaVan

**Affiliations:** 1 Departments of Pediatrics and of Microbiology and Immunology, Weill Medical College of Cornell University, New York, New York, United States of America; 2 Material Measurement Laboratory, National Institute of Standards and Technology, Gaithersburg, Maryland, United States of America; University of Pennsylvania, United States of America

## Abstract

We present a new antiviral strategy and research tool that could be applied to a wide range of enveloped viruses that infect human beings via membrane fusion. We test this strategy on two emerging zoonotic henipaviruses that cause fatal encephalitis in humans, Nipah (NiV) and Hendra (HeV) viruses. In the new approach, artificial cell-like particles (protocells) presenting membrane receptors in a biomimetic manner were developed and found to attract and inactivate henipavirus envelope glycoprotein pseudovirus particles, preventing infection. The protocells do not accumulate virus during the inactivation process. The use of protocells that interact with, but do not accumulate, viruses may provide significant advantages over current antiviral drugs, and this general approach may have wide potential for antiviral development.

## Introduction

Nipah (NiV) and Hendra (HeV) viruses are emerging zoonotic paramyxoviruses of the henipavirus family that cause fatal encephalitis in humans, with fatality rates of up to 75% [1]–[3]. Emerging zoonotic viruses are recently identified agents that can be transmitted from animals to humans and person-to-person; they often spread widely through animal hosts before infecting humans. Paramyxoviruses are single stranded RNA viruses that include a number of human pathogens. Henipaviruses share common structures such as a lipid envelope, receptor binding proteins (referred to as “G”) and fusion proteins (referred to as “F”). Direct transmission of NiV from its fruit bat (flying fox) mammalian reservoir to humans, and person-to-person transmission, allows infection to bypass the pig host which served to transmit the virus as it emerged into the human population[4]. HeV, also via a bat host, has caused disease in horses with transmission to horse-handlers and veterinarians, and since 1995 has caused sporadic illness in Australia [5]. NiV, in addition to acute infection, may cause asymptomatic infection in up to 60% of exposed people, and may lead to late-onset disease or relapse of encephalitis years after initial infection [6]. The vast geographic range of the fruit bat mammalian reservoir raises the possibility of wide spread of these human diseases, which presently have no approved clinical treatment or vaccine [7]. Other related human pathogens in the paramyxovirus family include parainfluenza viruses, respiratory syncytial virus (RSV), mumps and measles, all causes of significant human diseases affecting millions of children yearly.

The first steps in infection with paramyxoviruses are binding to the target cells, via interaction of the viral receptor-binding molecule (G for HeV or NiV) with the specific receptor molecules on the cell surface –EFNB2 (Ephrin-B2) for HeV, and both EFNB2 and EFNB3 for NiV, followed by the fusion of the viral envelope with the plasma membrane of the cell, a process mediated by the viral fusion protein (F). The receptor binding protein G, when engaged with receptor, “triggers” F to assume its fusion-ready conformation with its fusion domain exposed [8]. While F-activation is key for entry, the correct timing is critical. We suggest[9] that this timing of activation represents a target for intervention with infection, and that with the correct receptor mimic presented in the proper biological context, it is possible to induce triggering and to inactivate F, rendering the virus noninfectious.

Artificial cell-like particles, or protocells, bearing receptor mimics to attract and inactivate henipavirus envelope glycoprotein pseudotypes offer a new approach to preventing infection. To assess this strategy, we used quantitative assays for inhibitors of henipavirus infection that are based on envelope glycoprotein pseudotypes [10]. The pseudotypes bear the henipavirus entry molecules on a BSL2 agent (vesicular stomatitis virus - VSV) and use the identical entry mechanism as live henipaviruses[10]. Agents found to be inhibitory in this assay may either be those that block binding, or interfere with processing of F, or interfere with F activation and fusion. This strategy was used here to assess the inhibitory potential of this novel approach to anti-infectives: to determine whether synthetic protocells that bear the entry receptor for henipaviruses inactivate pseudotyped viruses.

### Development of artificial cells presenting membrane bound receptors (“protocells”)

**Figure 1:**
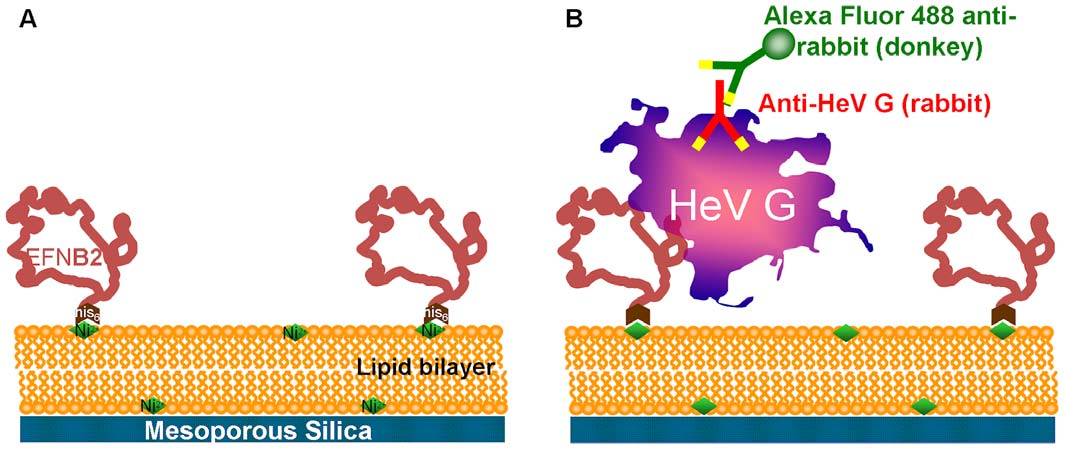
Scheme to produce synthetic protocells. a: planar or spherical nanoporous SiO_2_ surface with a lipid bilayer and EFNB2 binding protein. b: scheme used to confirm presence and activity of EFNB2 using purified Hendra G and primary and secondary antibodies.

Protocells have great potential to fight human disease [11] and serve as a platform to apply nanobiotechnology to treat disease [12], [13]. The protocells described here allow for biomimetic presentation of surface receptors in lipid bilayers that are supported on highly hydrophilic nanoporous silica particles. The construction of the protocells bearing henipavirus entry receptors is diagrammed in figure 1. Test protocells were prepared (shown in figure 1a) using 3 µm diameter nanoporous SiO2 particles following the methods of Brinker et al [14], [15]. Previous work has shown that lipid bilayers can be conveniently assembled over nanoporous silica surfaces and support the lipid bilayer in a fluid state analogous to the lipid bilayer supported on the actin cytoskeleton; nanoporous silica particles were selected to form the cell-like particles [16], [17] (Movie S1 shows confocal results of cell-like particles bearing EFNB2 in a red-fluorescent labeled lipid bilayer binding to the surface of engineered 3T3 cells presenting G and F; one of several control studies performed to confirm protocell synthesis). Nanoporous silica is viewed as biocompatible as silica is a micronutrient that will be absorbed in the body, forms of silica are used in food preparation and have been utilized for drug delivery [18]. It should be noted these particles are *not* crystalline silica – crystalline silica is associated with the occupational lung disease silicosis. A nickel chelating lipid bilayer was formed on the surface of the particles[19], [20]. Polyhistidine-tagged EFNB2 or EFNB1 (negative control; not a viral receptor) was then attached to the Ni2+ sites present in the lipid. Aliquots of purified soluble HeV G were incubated with the protocells; the presence of HeV G bound to the protocells was assessed by fluorescent microscopy and flow cytometry as shown in figure 1b. The protocells bearing EFNB2 efficiently bound HeV G and were fluorescent, while the protocells bearing EFNB1 did not bind HeV G, as expected.

### Protocells inactivate pseudotyped viruses

Protocells were incubated with pseudotyped viruses for one hour at 37°C and then the viral titer (as determined by viral entry on cultured human cells) was assessed and compared to the titer of untreated pseudotyped viruses. Individual protocells contained either EFNB1, EFNB2 or mixtures of the two; EFNB2 presenting protocells effectively inhibited entry by HeV pseudotyped virions, causing a dramatic decrease in viral titer after incubation with the protocells. The inhibitory effect of the protocells is specific for henipaviruses. Incubation with paramyxovirus human parainfluenza virus type 3, and with the rhabdovirus VSV, each of which uses a different entry receptor, did not result in a significant decrease in titer. In these experiments, pseudovirus infection was reduced 100% using the EFNB2 bearing protocells but VSV infection was reduced 11% when exposed to EFNB2 protocells (relative standard deviation is 13% for these measurements), confirming the specificity of inhibition.

**Figure 2:**
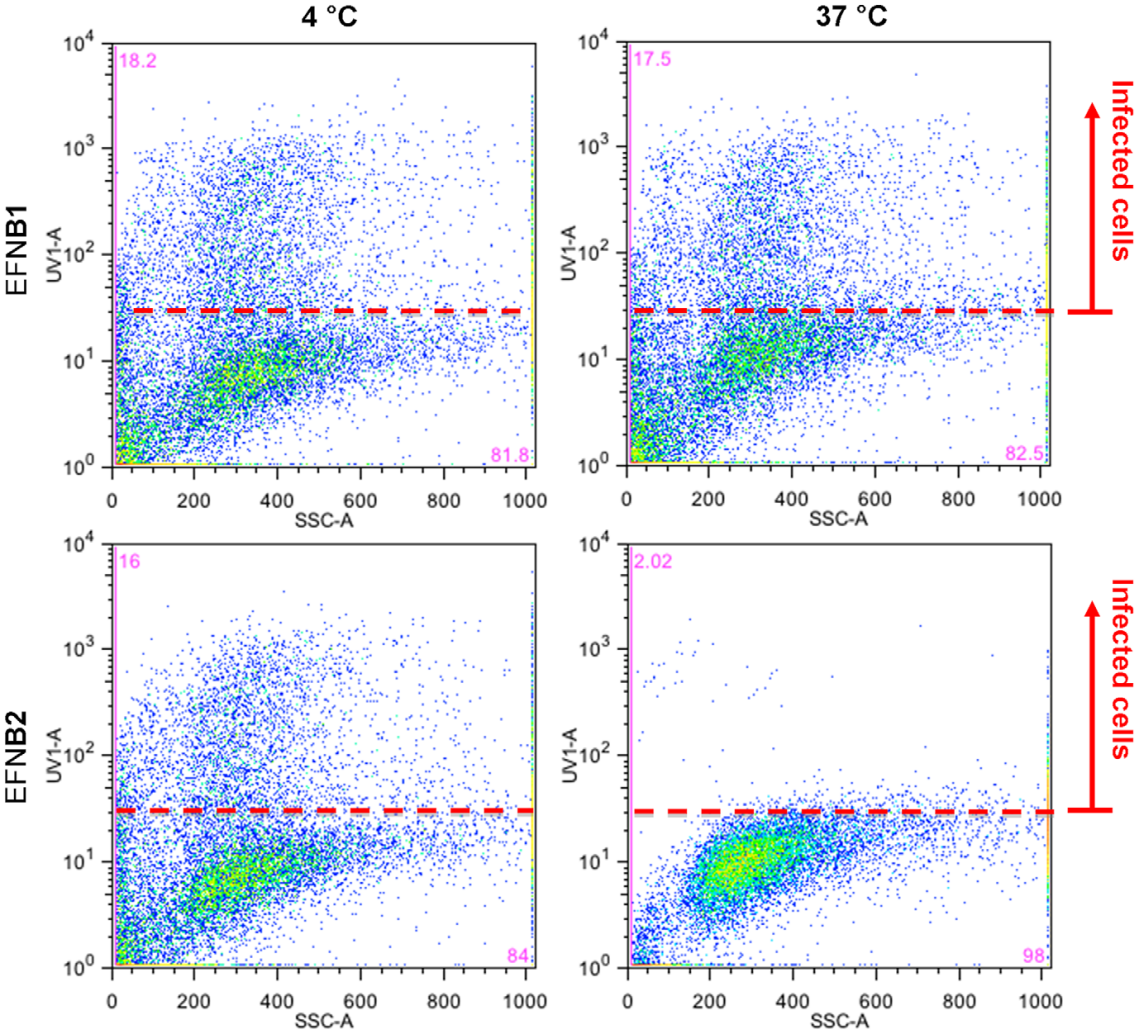
Inhibition of viral titer by protocells requires a 37°C step. Flow cytometry results showing cells infected with pseudoviruses after incubation with EFNB2 or EFNB1 bearing protocells at 4°C and 37°C. Data points falling above the line indicate that entry occurred and the cells expressed red fluorescent protein. FL1 is the fluorescent detector data that indicates measurement of a fluorescent label; SSC is side scatter data that indicates size. The location of the red line is determined by control experiments.

The inhibitory activity of EFNB2-bearing protocells is temperature dependent; infection of cultured cells is specifically inhibited after incubation at 37°C but not at 4°C (figure 2). In these experiments, pseudotyped viruses (hereafter called pseudovirus) were incubated with EFNB2 or EFNB1-bearing protocells at 4°C or 37°C, and then introduced to a culture with mammalian cells. In parallel, pseudoviruses were incubated with EFNB2-bearing sepharose beads at 4°C or 37°C. The sepharose beads provide an example of a presentation of receptor molecules in a static, non-biomimetic fashion, for comparison. Flow cytometry was carried out to determine the percentage of infected cells based on the expression of red fluorescent protein (RFP) gene incorporated into the pseudovirus. The cells that were infected by the pseudovirus are fluorescent; in each panel of figure 2, the top section represents the fluorescent cells counted in the flow cytometer. A control experiment with mock cells was run as part of each flow cytometer experiment to determine where the cut-off line should fall. In case of experiments to measure soluble G binding, the cut-off indicates fluorescent intensity indicative of binding; In the case of experiments to measure entry, the cut-off indicates fluorescent intensity associated with entry and expression of red fluorescent protein. Running mock cells before each experimental batch avoids falsely counting auto-fluorescence as a positive result. The raw flow cytometer plots display fluorescent intensity on axis (labeled UV1) versus an index of cell size from the side scatter detector (labeled SSC). Each dot on the plot is a single cell from the culture; each flow cytometer plot is therefore a comparison of a large cell population.

**Figure 3:**
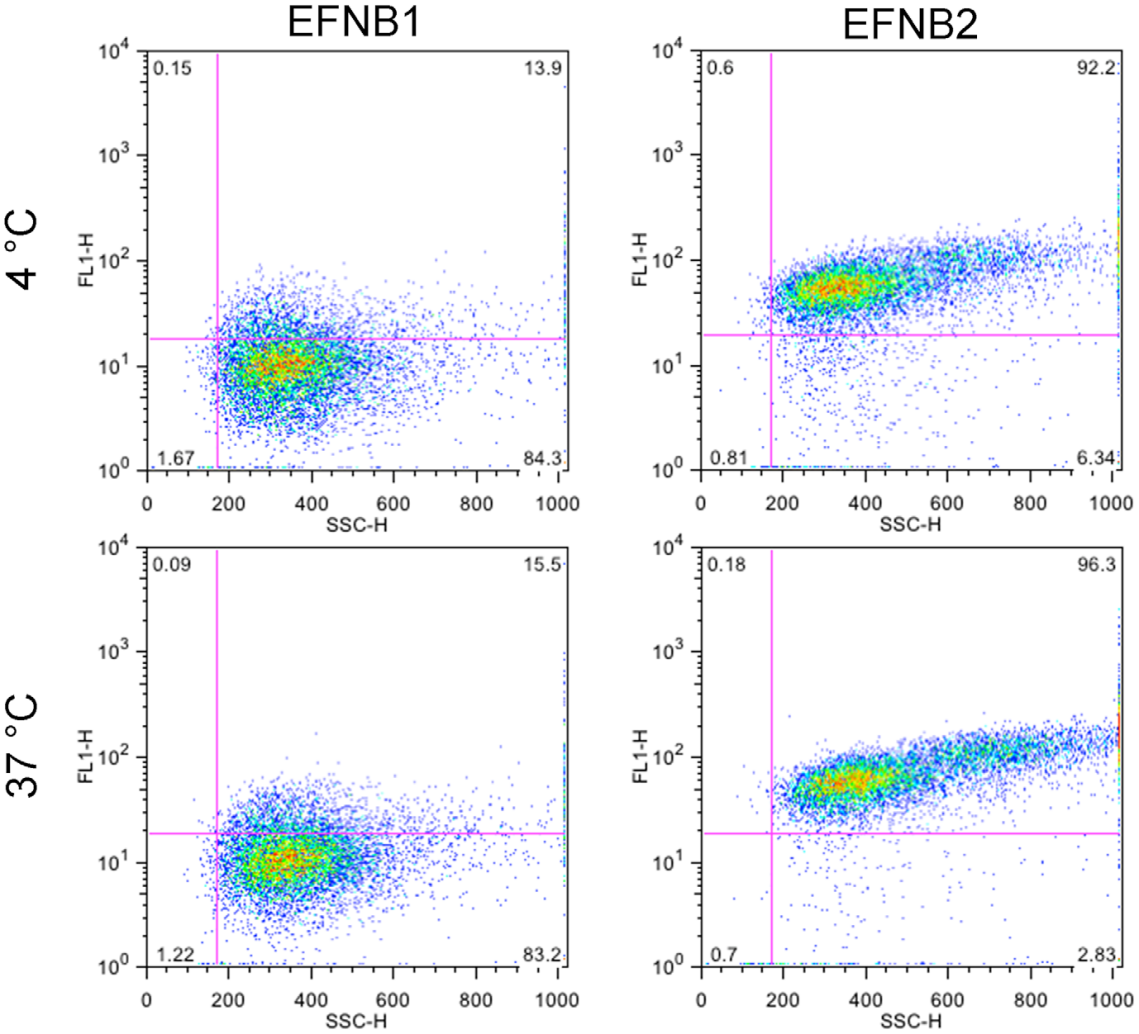
Binding of soluble Hendra G to EFNB1 and EFNB2 bearing protocells measured at 4°C and 37°C using flow cytometry. These plots show that soluble HEV G binding occurs equally to EFNB2 bearing protocells at these two temperatures and that there is little (not significant) soluble HEV G non-specific binding to the negative control protocells bearing EFNB1. FL1 is the fluorescent detector data that indicates measurement of the fluorescent label; SSC is side scatter data that indicates size.

In the lower right-hand panel of figure 2 (pseudovirus exposed at 37°C to EFNB2-bearing protocells before introduction of pseudovirus to mammalian cells) there is a drastic reduction in signal in the top section, indicating a reduction in the number of infected cells. For cells incubated with pseudovirus after exposure to the EFNB2-bearing protocells at 37°C, only 2.0% of cells were infected. The EFNB1-bearing protocells are not inhibitory and serve as negative controls with the same silica core and lipid bilayer, but a non-specific membrane protein. At the incubation temperature of 4°C – a temperature at which viral fusion machinery (F) is inactive – inactivation did not occur during the incubation with EFNB2-bearing protocells. For cells incubated with the pseudovirus exposed to EFNB2-bearing protocells at 4°C, the number of infected cells did not decrease, remaining approximately the same, at 18.2% and 16.0% (percentage of total cells exposed that became infected, for different conditions as shown in figure 2) respectively. In contrast, flow cytometry measurements of soluble G binding to EFNB2 bearing protocells was equal at 4°C and 37°C and soluble G did not bind to EFNB1 containing protocells (figure 3). These results show that inhibition by protocells occurs only at 37°C and not at 4°C and is not due to non-specific interactions, and that inactivation of infectivity by protocells, in distinction from binding to sepharose beads, entails a temperature-dependent step.

### The antiviral protocells do not accumulate virus

**Figure 4:**
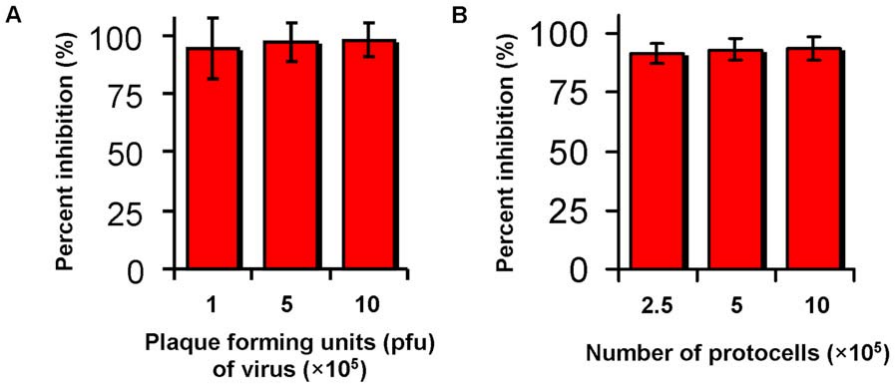
Inhibitory activity of protocells. a: Inhibitory effect is not saturated by increasing amounts of pseudovirus over this concentration range. Percent inhibition of viral titer by exposure to protocells bearing EFNB2, after addition of varying amounts of pseudovirus. b: Inhibitory effect is not saturated by decreasing the number of protocells over this concentration range. (Bars are mean ± one standard deviation, N = 3).

**Figure 5:**
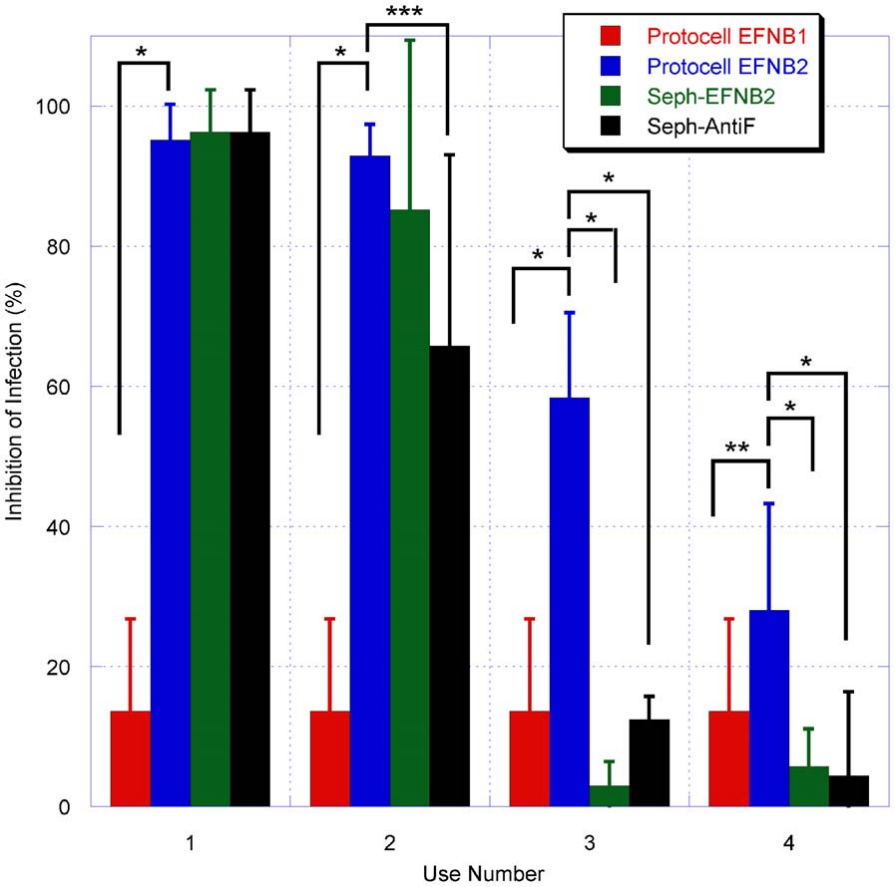
Inhibition of viral titer after repeated exposure of protocells bearing EFNB1 or EFNB2 along with sepharose particles bearing EFNB2 and sepharose particles bearing anti-F antibodies. For each repetition (1–4) the used protocells/particles were washed, pelleted, and re-incubated with fresh pseudovirus. The EFNB2-bearing protocells retain some inhibitory activity after four uses. * t-test for difference in means significant at 90% or greater confidence interval. ** t-test for difference in means significant at 88% confidence interval. *** t-test for difference in means significant at 84% confidence interval (Bars are mean ± one standard deviation. N = 3 except EFNB1-bearing protocells (negative controls) which were pooled with N = 12).

EFNB2-bearing protocells or, in parallel, sepharose beads bearing EFNB2, were incubated with pseudoviruses in concentrations ranging from 0.5 plaque-forming units (pfu) per protocell to 5 pfu/protocell. After incubation the supernatant fluid was collected and titered for infectious pseudoviruses. For the EFNB2-bearing protocells, activity vs. pseudoviruses was retained in our determination range, regardless of the concentration of virus (figure 4a) or concentration of protocells (figure 4b). To assess whether the protocells retain activity after repeated use, the EFNB2-bearing protocells and EFNB2-bearing sepharose beads (in parallel with protocells bearing EFNB1 and sepharose particles bearing anti-F antibodies) were washed, incubated with new pseudoviruses, and re-titered (figure 5). For each repetition (I–IV) the used protocells or particles were washed, pelleted, and incubated with new pseudoviruses. For the EFNB2-bearing protocells, activity vs. pseudoviruses was retained more strongly than the sepharose beads. The red bars in figure 4 show that EFNB2-bearing protocells retain significant inhibitory activity even at the fourth re-incubation. In contrast, as shown by the blue and green bars in figure 5, the efficacy of the sepharose beads bearing EFNB2 or anti-F antibodies had no inhibitory effect observed after the second use (t-test applied to means, each mean based on n = 3, 90% confidence intervals or higher). These data highlight a key feature of the protocells: they are reusable. Because of a larger number of binding sites on the sepharose beads, the comparison shown in figure 5 was biased in their favor – the sepharose beads contained roughly 10 times the number of ligands per bead as the protocells, evaluated with gel electrophoresis of EFNB2 removed from the beads and compared to lanes with EFNB2 of known concentration. Figure S1 shows typical gel electrophoresis results for EFNB1 and EFNB2 concentration removed from 20 µL aliquots of protocells and compared to known protein concentrations.

**Figure 6:**
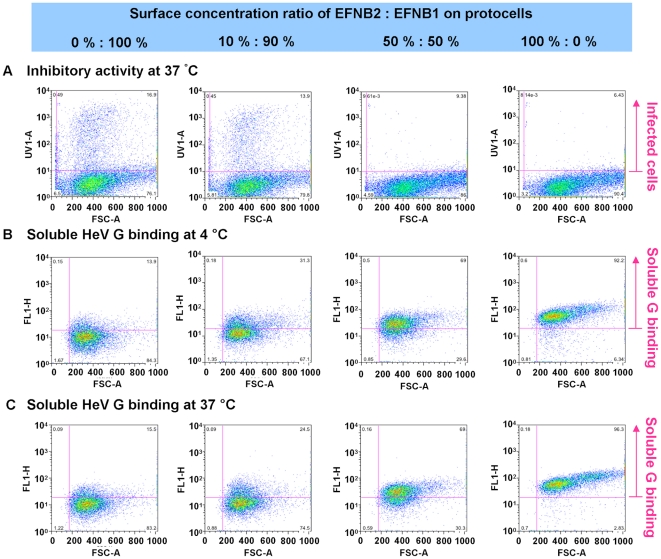
Inactivation of virus by protocells with mixtures of viral receptors and non-specific membrane proteins does not correlate with binding to soluble HeV G. a: inhibition of the infectivity of pseudoviruses after incubation with protocells bearing the indicated ratios of EFNB1: EFNB2 at 37°C. (No inhibition seen for experiments at 4°C) b: Binding of soluble HeV G to protocells at 4°C. c: Binding of soluble HeV G to protocells at 37°C. In panel a, the cells that have been infected are fluorescent and are shown in the top sections; in panels b and c, the protocells binding soluble HeV G are fluorescent, and are shown in the top sections. HeV G binding is equivalent at 37°C and at 4°C and scales with EFNB2 concentration.

To further assess whether inactivation is a consequence of binding, we asked whether there is any correlation between the surface concentration of EFNB2 on protocells and their effectiveness. For the measurements shown in figure 6, a panel of protocells were synthesized that bear different ratios of EFNB1 (not a receptor) and EFNB2 (receptor for G), ranging from 100% EFNB1 (0% EFNB2) on the left, to 0% EFNB1 (100% EFNB2) on the right. In panel (a), the protocells were incubated with pseudovirus at 37°C, and flow cytometry performed to quantify the inhibition of infectivity; in panel (a), the top section represents the fluorescent (infected) cells. In the experiments shown in panels (b) and (c), the same protocells were incubated with aliquots of purified soluble HeV G at 37°C (b) or at 4°C (c), and the presence of HeV G on the protocells was quantified by flow cytometry using fluorescently labeled antibodies. The protocells bearing the higher concentrations of EFNB2 efficiently bound HeV G and were fluorescent, while the protocells bearing the most EFNB1 did not bind HeV G, as expected, and binding increased corresponding to increasing ratios of EFNB2:EFNB1. Binding was equivalent at 37°C and at 4°C. Strong viral inactivation occurred with 50% EFNB2 at 37°C, even though binding of soluble HeV G was reduced due to the lower number of EFNB2. Likewise, little viral inactivation occurred at 4°C (see figure 2) though binding of soluble HeV G is equivalent at 37°C and at 4°C (panels 6b and 6c). The results suggest that the viral inactivation that occurs upon incubation with EFNB2-bearing protocells at 37°C, while it requires interaction with EFNB2 receptor, is not attributable to receptor binding alone, but requires an additional step. We propose that this step is the inopportune activation of F.

## Discussion

The antiviral protocells that we describe act specifically, effectively and renewably to inactivate pseudovirus, and do so *only* under conditions that permit triggering of the viral fusion protein. A temperature-dependent step is required for viral inactivation. Our hypothesis regarding the mechanism of inhibition by these protocells is that the protocells work as decoys; the virus interacts with the EFNB2 in the fluid surface of the particles, and then the F protein is triggered, rendering the virus inactivated. This hypothesis is supported by a number of observations: First, no virus particles or viral proteins have been identified bound to or sequestered within the protocells, despite repeated experiments (use of fluorescent antibodies, SEM examination and gel electrophoresis looking for viral proteins) which shows that inactivation is not related to simple G binding; the inactivation mechanism requires a temperature dependent step. F activation is one of two critical steps in this process that is temperature dependent. Second, while G-protein binding to the EFNB2 receptor is equal at 4°C and 37°C, the membrane fluidity at the lower temperature is reduced; reduced fluidity is the second critical feature that is temperature dependent. Decreased fluidity means an increased time for multiple copies of EFNB2 to accumulate near a virus, which introduces the temporal component to our hypothesis on the inactivation mechanism. The experiments performed with mixtures of EFNB1 and EFNB2 also support this hypothesis – as EFNB2 is sterically blocked on the membrane by EFNB1, the necessary signal can not be delivered in a short enough time frame. There is a narrow window in time during the virus interaction with the protocell for multiple EFNB2 receptors to engage the virus and cause F-protein triggering; if the necessary signal is not satisfied, the virus appears to disengage from the protocell and F-protein is not triggered.

There is urgent need for new, effective antiviral strategies for paramyxoviruses, including the pediatric respiratory viruses parainfluenza and respiratory syncytial virus, as well as the emerging henipaviruses and orthomyxoviruses. Acute respiratory infections cause ≈20% of global mortality in children under five years of age, and pediatric respiratory viruses in the paramyxovirus family (parainfluenza virus, respiratory syncytial virus) account for a major portion of this disease. This study presents a radical new antiviral strategy, which could be applied to a wide range of viruses that infect human beings via membrane fusion. If efficient receptor-mimics can trigger and thus inactivate viral fusion machinery after budding and release, but before contact with the next target cell, then released virus could be rendered noninfectious and viral spread halted. This mechanism is of clinical relevance since therapy for encephalitis, pneumonia and other diseases caused by paramyxoviruses and other enveloped viruses will often need to be effective when used after the onset of infection.

The need for entirely new antiviral approaches is especially urgent in light of recent evidence that the development of resistance to antiviral drugs may be rapid. The global emergence of influenza viruses resistant to oseltamivir has highlighted the importance of developing new antiviral approaches that target different portions of the viral infection machinery [21]. We suggest that the design of this novel strategy in which the viral fusion mechanism is “prematurely” triggered makes it unlikely to elicit resistant viruses, since viruses that escape the effects of this strategy will be overly handicapped. Mutant viruses with alterations in their fusion proteins that could emerge under selective pressure would have impaired fusion machinery triggering mechanisms, and should be far less fit and transmissible than the parent viruses. No virus whose fusion mechanism has triggered prematurely will be able to infect, and no virus whose fusion machinery is not triggerable will be able to infect. We propose that this new strategy is thus highly unlikely to be rendered ineffective by viral resistance. The approach we describe here may have wide potential for antiviral applications, and the use of protocells may provide significant advantages over available approaches.

## Methods

### Cells and viruses

293T (human kidney epithelial) and Vero (African green monkey kidney cells) were grown in Dulbecco's modified Eagle's medium (DMEM) (Mediatech; Cellgro) supplemented with 10% fetal bovine serum and antibiotics in 5% CO2.

### Plasmids, reagents and transient expression of G and F

HeV wild type G and F in pCAGGS were a gift from Lin-Fa Wang. To generate the shortened cytoplasmic tail variant of HeV G (HeV G-CT32 [22]), an internal primer containing an EcoR1 site and initiating at position 32 of the open reading frame was used for nested PCR. The primer sequence was: 5′ GGAATTCGGCACAATGGACATCAAG 3′. To generate a soluble form of HeV G, the extracytoplasmic domain of G was subcloned into pSEC-tag (Invitrogen) [23]. Transfections were performed according to the Lipofectamine 2000 reagent manufacturer's protocols (Invitrogen).

### Rabbit polyclonal antibodies

Anti-HeV G and anti-HeV F polyclonal antibodies were raised in rabbit by DNA immunization of HeV G or HeV F (Genovac).

### Pseudovirus infection assay

The VSV-ΔG-RFP is a recombinant VSV derived from the cDNA of VSV Indiana, in which the G gene is replaced with the Ds-Red (RFP) gene. Pseudotypes with HeV F and G were generated as described [7], [23], [24]. Briefly, 293T cells were transfected with plasmids encoding either VSV-G, HeV-GCT32/F, HeV-GCT32, or HeV-F. 24 hrs post-transfection, the dishes were washed and infected (m.o.i. of 1) with VSV-ΔG-RFP complemented with VSV G. Supernatant fluid containing pseudovirus (HeV F/CT32-G or VSV-G) was collected 24 hrs post-infection and stored at −80°C. For infection assays, HeV F/CT32-G or VSV-G pseudotypes (controls) were used to infect Vero cells. Various protocells or control agarose beads were added at various times and concentrations as indicated in the figures. RFP production at 36 h was analyzed by fluorescent microscopy[25] and flow cytometry.

### Flow cytometry

Flow cytometry was performed on a BD FACS Vantage SE with DiVa upgrade (BD Biosciences, San Jose, CA) equipped with a Stabilite 2017 argon laser turned to 488 nm and a BeamLok 2060 argon-krypton laser turned to 568 nm, both from Spectra-Physics (Mountain View, CA) at the Hospital for Special Surgery. For RFP registration, the cells were excited with 568 nm and the fluorescence of RFP emission was detected with a BP610/20 nm filter.

### Protocell Reagents

Nominal 10 µm and 3 µm mesoporous silica microparticles were purchased from GFS, each with nominal 10 nm internal pore size. POPC and a nickel-chelating lipid, the nickel salt of 1,2-dioleoyl-sn-glycero-3-{[N(5-amino-1-carboxypentyl)iminodiacetic acid] (DOGS-NTA-Ni), were purchased from Avanti Polar Lipids (Alabaster, AL) and dissolved in chloroform to form solutions of 10 mg/mL and 1 mg/mL, respectively. Red fluorescent dye, 1,2- dihexadecanoyl-sn-glycero-3- phosphoethanolamine, triethylammonium salt (Texas-red DHPE), was purchased from Invitrogen. His-tagged EFNB2 and EFNB1 was purchased from Sigma-Aldrich and hydrated following the manufacturers protocol. Alexa Fluor 488 donkey anti-rabbit IgG was purchased from Sigma-Aldrich. Dilute phosphate buffered saline (PBS) for washing and formation of lipid vesicles was made by diluting 100 mL cell-culture grade 1X PBS with 400 mL of deionized (DI) water.

### Mesoporous silica cores

Commercially purchased mesoporous silica microparticles (GFS) were treated to form a hydrophilic surface before use. The particles were stirred for 10 min at 85°C in 4% hydrogen peroxide and 4% ammonium hydroxide, in water, followed by repeated centrifugation and washing with DI water, stirring for 10 min at 85°C in 4% hydrogen peroxide and 0.4 mol/L hydrochloric acid (HCl), in water, followed again by repeated centrifugation and washing with DI water. The final particle concentration was adjusted to ≈107 particles/mL.

### Unilamellar lipid vesicles

POPC dissolved in chloroform (to 10 mg/mL) was mixed with 5% of 1 mg/mL 1,2-dioleoyl-sn-glycero-3-{[N(5-amino-1-carboxypentyl)iminodiacetic acid] (DOGS-NTA-Ni, Avanti Polar Lipids, Alabaster, AL) and 1% fluorescent Texas-red DHPE (in selected experiments to confirm lipid presence, Invitrogen) in a glass vial (dye was included in selected experiments to confirm lipid presence). The solvent was evaporated using a nitrogen stream until dry and the vial was kept under vacuum for 1 h to 2 h to remove residual solvent. The lipid film was hydrated using deionized (DI) water overnight at 4°C. The final lipid concentration was 2 mg/mL. The hydrated lipids were vortexed for several minutes and were extruded using a mini-extruder (Avanti Polar Lipids) with 0.1 µm polycarbonate membrane filters (Whatman, Inc., Newton, MA). The lipid solution was passed through the extruder at least 19 times and diluted with PBS to 1 mg/mL.

### Lipid coating of protocells

A 1 mL aliquot of silica particle suspension was centrifuged, the supernatant was removed, and 1 mL of the freshly prepared small unilamellar vesicle solution was added and incubated with shaking for 45 min to obtain silica particle supported lipid bilayers. The final mixture was repeatedly centrifuged and washed in dilute PBS to remove excess lipid.

### Addition of receptor moieties to protocells

Lipid-coated particles were incubated with 50 µL of 100 µg/mL of his-tagged EFNB2 (or EFNB1 for control samples, Sigma-Aldrich) in PBS at room temperature for 30 min followed by repeated centrifugation and washing in PBS to remove free protein. The 3 µm diameter protocells have approximately 104 copies of the receptor protein on each particle after the completion of synthesis.

### Polymer beads

Highly cross-linked 6% agarose polymer microparticles (Chelating Sepharose Fast Flow, Amersham Biosciences) were used as controls to produce particles with EFNB1 and EFNB2 attached to the surface via a linker. The particles were charged with Ni2+ ions and were used following the manufacturers protocols.

## Supporting Information

Figure S1 **Image of a typical gel electrophoresis experiment to evaluate quantity of EFNB1 and EFNB2 incorporated into protocells.** Left Panel: EFNB1 isolated from 20 µL aliquot of EFNB1 bearing protocells compared to molecular weight ladders and known quantities (1 µg, 0.2 µg, and 0.04 µg) of purified EFNB1. Right Panel: EFNB2 isolated from 20 µL aliquot of protocells compared to molecular weight ladders and known quantities (1 µg, 0.2 µg, and 0.04 µg) of purified EFNB2. In each case, the conclusion is that the 20 µL aliquot of protocells contains approximately 0.2 µg of EFNB1 or EFNB2, respectively.
(TIF)

Movie S1 **Movie showing rotation of three dimensional image based on three dimensional deconvolution of fluorescent confocal z-stack images.** The experiment consisted of engineered HeLa cells expressing HeV G and F on their membrane interacting with EFNB2 bearing protocells to evaluate the bilayer formation on the protocells and protocell - viral binding moiety interactions. The video shows well formed lipid layers wrapped around each protocell and also shows the protocells binding to cell membranes. In this reduced system, like the experiments with soluble G binding to protocells and analyzed with flow cytometry, simple binding does occur between EFNB2 bearing protocells and cells membranes' expressing HeV G and F. However, there is neither internalization of the protocells nor fusion of the protocells and cell membranes. The HeLa cells were expressing green fluorescent protein in the cytoplasm for imaging; the protocells were synthesized with red fluorescent lipid for imaging.
(AVI)
