# Supplementary figures and images for: Synthetic Protocells Interact with Viral Nanomachinery and Inactivate Pathogenic Human Virus
Source: PLoS One. 2011 Mar 1;6(3):e16874. doi: 10.1371/journal.pone.0016874 (PMC3046955; doi:10.1371/journal.pone.0016874)

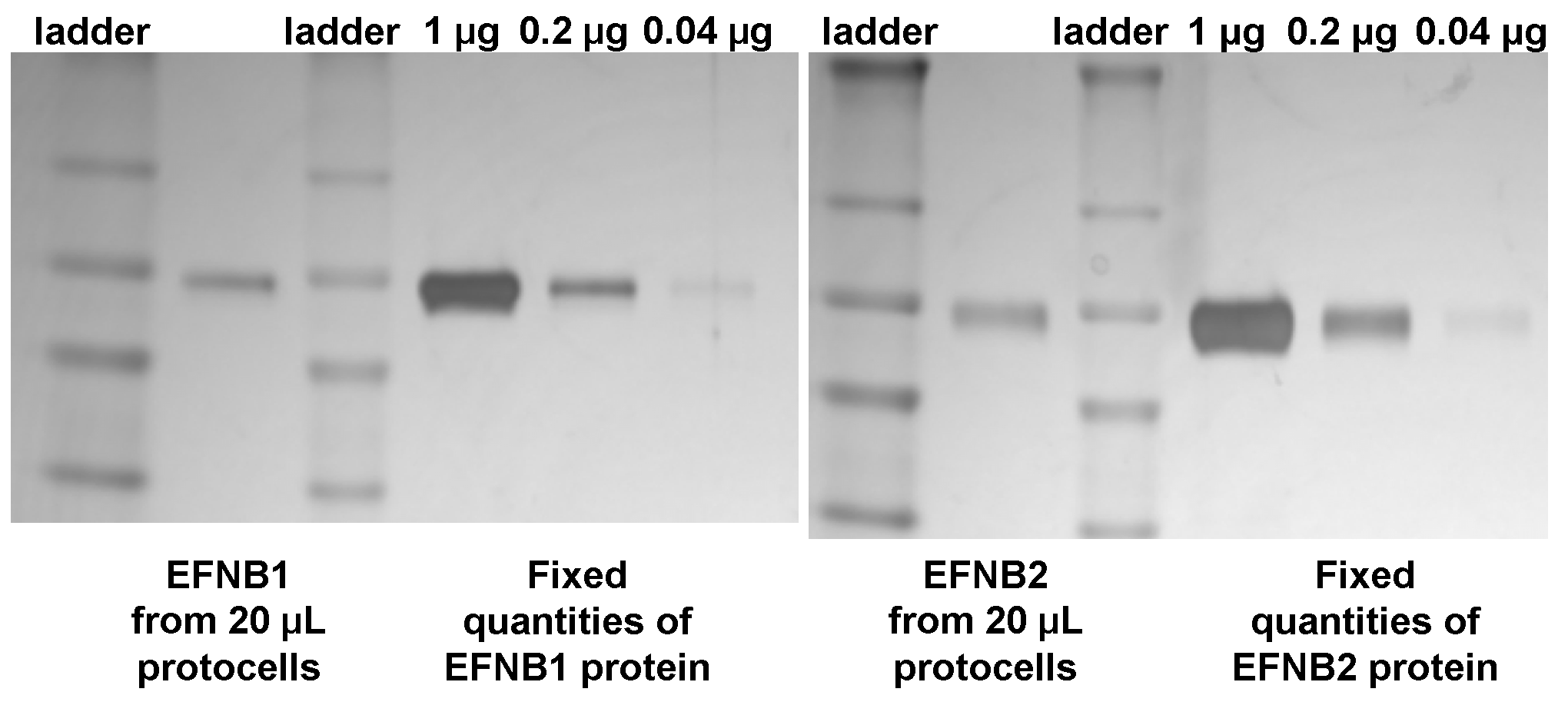

Supplement: Figure S1 — Image of a typical gel electrophoresis experiment to evaluate quantity of EFNB1 and EFNB2 incorporated into protocells. Left Panel: EFNB1 isolated from 20 µL aliquot of EFNB1 bearing protocells compared to molecular weight ladders and known quantities (1 µg, 0.2 µg, and 0.04 µg) of purified EFNB1. Right Panel: EFNB2 isolated from 20 µL aliquot of protocells compared to molecular weight ladders and known quantities (1 µg, 0.2 µg, and 0.04 µg) of purified EFNB2. In each case, the conclusion is that the 20 µL aliquot of protocells contains approximately 0.2 µg of EFNB1 or EFNB2, respectively. (TIF) [file pone.0016874.s001.tif]
